# Investigation on the Hydrate Blockage Avoidance Performance of Two Anti-Agglomerants and Their Mixture with PVP

**DOI:** 10.3390/molecules30020308

**Published:** 2025-01-14

**Authors:** Sunan Wang, Litao Chen, Lei Guo, Jiansheng Luo, Liangliang Ren, Xiong Xiang, Tie Geng, Changhong Yu, Zilong Meng

**Affiliations:** 1 Oilfield Chemical Division, China Oilfield Services Limited, Sanhe, Langfang 065201, China; wangsn@cosl.com.cn (S.W.); guolei5@cosl.com.cn (L.G.); luojsh2025@163.com (J.L.); renll4@cosl.com.cn (L.R.); xiangxiong@cosl.com.cn (X.X.); gengtie@cosl.com.cn (T.G.); 2 School of Petroleum Engineering, China University of Petroleum (East China), Qingdao 266580, China; yuchanghong13@163.com (C.Y.); zilong.meng@celanese.com (Z.M.)

**Keywords:** hydrate, blockage avoidance, anti-agglomerants, PVP, subcooling

## Abstract

The hydrate blockage avoidance performance of two anti-agglomerants (coconut amidopropyl dimethylamine, propylene bis (octadecylamidopropyl dimethylammonium chloride)) and their mixtures with polyvinylpyrrolidone (PVP) was tested in a high-pressure rocking cell apparatus. The effect of gas–liquid ratio, water content and PVP concentration were analyzed. A method for evaluating the kinetic inhibiting and anti-agglomerating performance of hydrate inhibitors was established. It was found that coconut amidopropyl dimethylamine had good anti-agglomerating performance at a low gas–liquid ratio (0.5) and various water content levels (20~80%), while propylene bis (octadecylamidopropyl dimethylammonium chloride) had a good anti-agglomerating performance only at a low gas–liquid ratio (0.5) and high water content (80%), and the hydrate volume fraction was up to 23.27% for good anti-agglomeration. When PVP was mixed with the above two anti-agglomerants, it was found that coconut amidopropyl dimethylamine could significantly reduce the kinetic inhibition performance of PVP, while propylene bis (octadecylamidopropyl dimethylammonium chloride) had no significant effect on the kinetic inhibition performance of PVP. The maximum subcooling was 4.4 °C. PVP had no significant effect on the anti-agglomerating performance of the two anti-agglomerants, and the maximum hydrate volume fraction was 18.87% when the agglomeration was well inhibited.

## 1. Introduction

Gas hydrate is a kind of cage-like compound formed by trapping gas molecules in a water network at a suitable pressure and temperature [1]. During deepwater oil and gas drilling and extraction, it is very easy to form hydrates in the wellbore or pipeline, causing blockage and leading to huge economic losses [2,3]. The inhibitor injection-based strategy is often used in production to avoid hydrate blockage. Methanol and ethylene glycol are traditional thermodynamic inhibitors which could change the hydrate equilibrium conditions (move the hydrate formation boundaries) with high reliability but in high dosage. In recent decades, low-dosage hydrate inhibitors (LDHIs) have tended to be widely used, which can effectively inhibit hydrate formation or/and agglomeration at low dosage (<1 wt%, methanol or glycol dosage is usually higher than 30%), prevent hydrate blockage, and have a good economic saving. The main categories of LDHIs are anti-agglomerants (AAs) and kinetic hydrate inhibitors (KHIs) [1]. AAs allow large amounts of hydrates to form but prevent them from aggregating into large blocks, while KHIs act at the hydrate nucleation and growth stages by the preventing the formation of large amounts of hydrates [4].

KHIs are novel and economical hydrate inhibitors, which have been developed since the 1990s. KHIs make it difficult to form hydrate blockages by reducing the nucleation kinetics of hydrates, prolonging the induction period of hydrates, or even preventing the hydrate crystals from growing to critical sizes. At present, most of the KHIs studied are polymers containing amide groups, which are divided into two categories: cyclic amide groups and chain amide groups. Typical cyclic amide-based polymers include pentacyclic polyvinylpyrrolidone (PVP) [5,6,7], hexacyclic polyvinylpiperidone (PVPip) [7,8], heptacyclic polyethylene caprolactam (PVCap) [9,10], and octacyclic polyvinylazetoxanone (PVACO) [11]. The inhibition performance of cyclic amide polymers is directly proportional to the size of the lactam ring [12]. The N-C=O group on the cyclic amide ring forms a new hydrogen bond with the hydrate cage water molecules, destroying the cage structure. The side chain of chain amide-based polymers contains active functional groups, which could combine with the hydrogen atom of water molecules via hydrogen bonding. On the one hand, it could destroy the order of water molecules, and on the other hand, it could destroy the hydrogen bonds in the hydrate cages. At the same time, the chain polymer could adsorb on the surface of hydrate crystal and inhibit its further growth. Polymers containing amino groups include polyalkyl acrylamide, polydialkyl acrylamide, polyethyleneamine, polypropyl amine, polymaleimide, etc. At present, it is believed that there are three main acting mechanisms of KHI: the disturbance mechanism that inhibits hydrate nucleation, the adsorption mechanism that inhibits hydrate growth, and the layer mass transfer obstruction mechanism. The disturbance mechanism focuses on the disruption of the order of water molecules, which delays the nucleation of hydrates [13,14]. The adsorption mechanism focuses on the interactions of inhibitor molecules and the hydrate crystal surface. The adsorption of inhibitors on the hydrate surface occupies the nucleation sites, thereby hindering the rapid hydrate growth [10,15]. The mechanism of layer mass transfer focuses on the effect of inhibitor on gas–liquid mass transfer, which forms a barrier layer on the surface of hydrates, increasing the mass transfer resistance, and thereby inhibiting the growth of hydrates [16,17,18]. In fact, the KHIs may cause an effect by using the disrupting, adsorbing, and hindering mass transfer mechanism all together, at the same time. Before the nucleation, the interaction between inhibitor molecules and water molecules may affect their order and make it difficult to form a cage structure. After nucleation, the KHI molecules could adsorb on the hydrate surface to produce steric hindrance. This mass transfer resistance will gradually increase with the increase in inhibitor molecules on the hydrate surface. In the hydrate growth stage, the hydrate growth could be suppressed if enough KHI molecules adsorb on the surface. Although KHIs could effectively prevent hydrate formation, it could only work for a certain period of time. A disastrous hydrate growth usually occurs once the KHI fails. Therefore, the reliability of KHI has always been the biggest concern.

Once KHI fails, a massive hydrate will form. In that case, AAs could provide a second layer of protection. AAs could help to disperse the hydrate in the oil and/or water to form a stable slurry. The hydrate slurry could not only prevent the wellbore or pipeline blockage, but also improve the transporting efficiency. Quaternary ammonium cationic surfactants are a commonly used class of AAs. They usually contain long alkyl chains, such as n-butyl or n-amyl, which could form a good anti-agglomeration layer at the hydrate–oil interface, thereby preventing the agglomeration of hydrate particles [19]. Some quaternary ammonium salts, such as bis-hexadectdimethylammonium chloride and n-dodecyltributylammonium bromide, have good anti-agglomerating properties. Delroisse [20] found that the water-soluble quaternary ammonium salt DA50 has good anti-agglomerating performance at low water cut. York et al. [21,22,23] found that the combined effect of quaternary ammonium salts with NaCl and MgCl_2_ on structure I hydrates was good in a series of rocking cell experiments. The quaternary ammonium salts also had a good anti-agglomerating effect on structure II hydrates in a solution containing 0.5 wt% methanol. A series of surfactants, alkyl glycosides and CWPUUs, rhamnolipids, lauramidopropyl dimethylamine, lecithin, lanolin, vitamin E succinate are all publicly reported as anti-agglomerants. Non-cationic epiactivators, such as rhamnolipids [24] and lauramide propyldimethylamine, [25] have good anti-agglomerating performance in oil–water emulsions with low water cut (50%). Rhamnolipids are biosurfactants with diverse structures, which have the effects of emulsifying, demulsifying and reducing surface/interfacial tension, and pose little harm to the environment [25,26,27]. York et al. [21] demonstrated for the first time that rhamnolipids could prevent cyclopentane hydrate from agglomerating. Sun et al. [25] found that cocamidopropyl dimethylamine was effective in avoiding hydrate agglomeration at 0–100% water cut. It indicated that anti-agglomeration could not be equivalented with emulsions, explaining why some high-efficiency emulsifiers could not prevent hydrate plugging, while some demulsifiers, such as Dowfax DM655, could act as hydrate anti-agglomerants. Lecithin and lanolin are taken from animals/plants and wool, respectively. They both have a strong emulsifying effect, which can reduce the oil–water interfacial tension and promote the dispersion of hydrates in a liquid. It was reported that the anti-agglomerating performance of lecithin and lanolin are better than those of Span80 in high water cut (>50%) systems [26]. Vitamin E succinate is a derivative of vitamin E, which forms stable micelles in water and has a good anti-agglomerating effect on hydrates [27]. Although AAs are effective even at high hydrate concentrations, there are also drawbacks. The viscosity of the liquid phase will inevitably increase as the slurry forms. The requirement of sufficient liquid to form slurry may also limit its application in low-liquid loading systems, such as gas pipelines.

KHIs and AAs have different mechanisms and effects, each with their own advantages and disadvantages. If KHIs are used in combination with AAs, they may be able to complement each other’s strengths and improve the reliability of blockage control. A two-layer protection hydrate inhibitor, containing both KHI and AA, may be more promising. However, what is the effect of KHIs on the performance of AAs, and what is the effect of AAs on the performance of KHIs? Answering this question requires specific studies. In order to provide some understanding on the mutual influence of AA and KHI in their inhibiting performance, in this paper, the hydrate blockage inhibiting performance of two AAs (containing amidopropyl dimethylamine, ammonium) and their mixture with PVP, were investigated using high-pressure rocking cell experiments, and the subcooling degree, induction time and blocking grades of the inhibitors were determined for different gas–liquid ratios, water contents and PVP concentrations. The results could provide insights for clarifying the action characteristics of LDHIs and exploring efficient inhibitor formulations.

## 2. Materials and Methods

### 2.1. Materials and Apparatus

**Figure 1:**
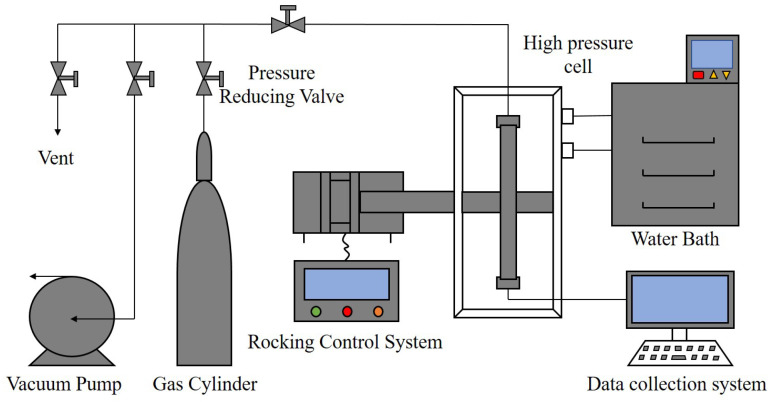
Schematic of the high pressure rocking cell apparatus.

The specifications and sources of the chemicals used in the experiments are given in Table 1. The two AAs (AA1 and AA2) used are amide surfactants. Figure 1 is a schematic diagram of the high-pressure rocking cell apparatus. Notably, 316L stainless steel with good corrosion resistance was used as the body material of the reaction cell, which can withstand the pressure of 30 MPa. In addition to the reaction cell, the rocking cell apparatus also includes a rocking system, a gas injection system, a temperature control module and a data acquisition module. The rocking system is composed of a motor, a drive, a rotating rod and a control cabinet. The rocking frequency was set as 1 time/min. The rocking angle is ±90°. The data acquisition system consists of all the temperature, pressure and slider position transducers and real-time data acquisition software. The full measuring range of the temperature sensor is −50 °C to 100 °C, with an accuracy of ±0.1 K, while the full measuring range of the pressure sensor is 0–40 MPa, with the accuracy of ±0.01 MPa. The operating temperature range of the slider movement sensor is −40 °C to 75 °C. The linearity is less than ±0.02% of the full scale. The data acquisition software collects 4 sets of data per second to reflect the falling process of the slider. The gas injection system is composed of a methane gas cylinder with a pressure regulator, with the ubing, valve and vaccum pump. A chiller (Ningbo Tianheng THX-2030H, Tianheng, Ningbo, China) was used to control the temperature of the water bath. The cooling rate was set to ~2 °C/h. An electronic balance (BSM-420.3, Shanghai Zhuojing Electronic Technology Co., Ltd., Shanghai, China) with an accuracy of ±0.001 g was used to weigh the mass of the chemicals.

### 2.2. Experimental Procedure

The rocking cell apparatus was used to conduct experiments to evaluate the performance of KHI and AA on methane hydrate in water–oil–gas systems. The slider position sensor was employed to acquire the slider movement information to characterize the hydrate agglomerating status in the cell. In this case, the anti-agglomerating performance of AAs on hydrates was evaluated. The kinetic inhibiting parameters, such as the subcooling degree and the induction time, were measured by the constant rate cooling method. The effect of AA/KHI on hydrate nucleation was measured. The specific procedures in experiments are listed as follows: (1) confirm the motor is powered off, clean the reactor cell by using petroleum ether and dry it with compressed air; (2) prepare the liquid sample with No. 5 white oil, deionized water and AA/KHI, and add it into the reaction cell, seal the reactor cell and vacuum it to −0.1 MPa, turn on the chiller and set the temperature to be 16 °C; (3) when the water bath temperature is stable, charge the reactor cell with methane gas to the desired pressure; (4) turn on the motor to rock the reactor cells, the methane gas dissolution is accelerated indicated by rapid pressure decrease; (5) when the pressure in the reactor cell is stable, recharge the cell with methane gas to ~8 MPa, set the chiller temperature to 0 °C with a cooling rate of ~2 °C/h, and hydrate formation starts when indicated by a pressure decrease; (6) when the pressure decrease stops, hydrate formation is considered to be complete, stop the experiment, vent the gas and clean the reactor cell; (7) repeat steps (1) to (6) for experiments for other chemicals.

### 2.3. Hydrate Volume Fraction Calculations

Hydrate volume fraction is a characterization of the volume percentage of hydrates in a liquid phase. As hydrate formation occurred, the slider movement can be significantly affected by the hydrate volume fraction. Comparing the slider movement at various hydrate volume fractions for different AAs/KHI, the anti-agglomerating performance could be quantitatively characterized. Therefore, calculation of the hydrate volume fraction is necessary.

The equation for calculating the hydrate volume fraction *φ_hyd_* was expressed as follows [28,29]:

(1) $$\varphi_{\mathrm{hyd}}=\frac{V_{\mathrm{hyd}}}{V_{\mathrm{hyd}}+V_{\mathrm{oil}}+(V_{\mathrm{water}}-V_{\mathrm{water, conv}})}$$

where *V_hyd_* represents the hydrate volume in liquid, mL; *V*_oil_ represents white oil volume, mL; *V_water_* represents initial water volume in absence of hydrate, mL; *V_water_*_,_*_conv_* represents the volume of water converted to hydrate, mL.

The hydrate volume could be calculated as:

(2) $$V_{hyd}=\frac{m_{hyd}}{\rho_{hyd}}$$

where *ρ_hyd_* represents the methane hydrate density, 0.91 g·mL^−1^; *m_hyd_* represents hydrate mass, g.

(3) $$m_{\mathrm{hyd}}=M_{\mathrm{hyd}}\cdot n_{\mathrm{hyd}}$$

where *n_hyd_* represents methane hydrate amount, mol; *M_hyd_* represents methane hydrate molar mass, which is 124 g/mol. Herein, the methane hydrate formula was assumed as CH_4_·6H_2_O. The hydrate mass *m_hyd_* and the water consumption *V_water_*_,_*_conv_* could be calculated from gas consumption.

During hydrate formation, the solubility of methane also changes due to the change in pressure and temperature. Because the methane solubility in water is very small, only the methane solubility in white oil was taken into account. According to the mass balance equation, the methane consumption amount during hydrate formation could be obtained:

(4) $$n_{\mathrm{gas},1}+n_{\mathrm{oil},1}=n_{\mathrm{gas},2}+n_{\mathrm{oil},2}+n_{\mathrm{hyd}}$$

where *n_gas_*_,1_ represents the gaseous methane amount before hydrate formation, it could be determined according to the equation of state, mol; *n_gas_*_,2_ represents the gaseous methane amount during hydrate formation, mol; *n_oil_*_,1_ represents the methane amount dissolved in white oil before hydrate formation, mol; *n_oil_*_,2_ represents the methane amount dissolved in white oil during hydrate formation, mol.

*n_gas_*_,1_, *n_gas_*_,2_ was calculated by using Patel–Teja equation of state:

(5) $$P_{1}V_{\mathrm{gas}}=Z_{1}n_{\mathrm{gas},1}RT_{1}$$

(6) $$P_{2}V_{\mathrm{gas}}=Z_{2}n_{\mathrm{gas},2}RT_{2}$$

where *V* is the volume of gas phase, m^3^; *R* denotes general gas constant, 8.314 J·mol^−1^·K^−1^; *P*_1_ and *P*_2_ represent system pressures before and after hydrate formation, respectively, Pa; *T*_1_ and *T*_2_ represent system temperatures before and after hydrate formation, K; *Z*_1_ and *Z*_2_ represent compression factors of methane gas before and after hydrate formation, respectively.

The methane solubility in white oil was measured and regressed to the following correlations:

(7) $$n_{\mathrm{oil},1}=\frac{3\times 10^{-5}P_{1}^{3}-0.001P_{1}^{2}+0.0273P_{1}-0.0029}{100}\times V_{\mathrm{oil}}$$

(8) $$n_{\mathrm{oil},2}=\frac{3\times 10^{-5}P_{2}^{3}-0.001P_{2}^{2}+0.0273P_{2}-0.0029}{100}\times V_{\mathrm{oil}}$$

## 3. Methods for Evaluating Inhibitor Performance

### 3.1. Anti-Agglomeration

**Figure 2:**
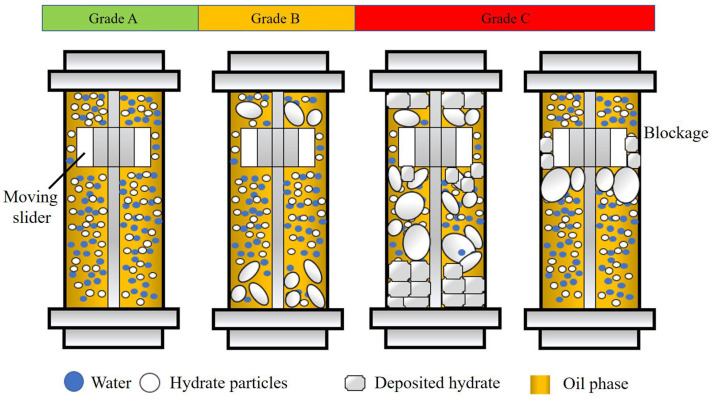
Schematic on the classification of the anti-agglomerating performance of AA/KHI.

**Figure 3:**
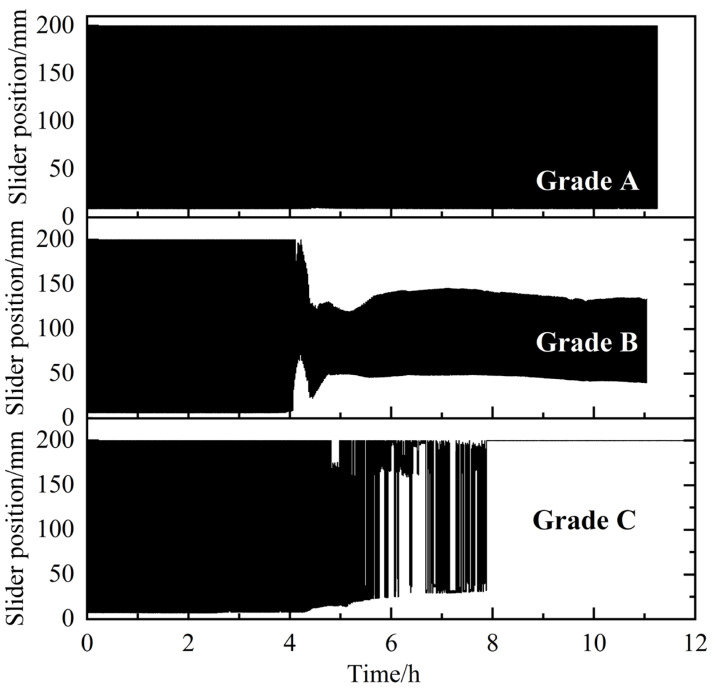
Schematic on the trajectory of the slider movement for the three classes of AAs [28].

An evaluation and classification method for AAs was established based on the characteristics of the rocking cell apparatus, as shown in Figure 2. The formation and agglomeration of hydrates directly affects the motion state of the slider. A large hydrate aggregate may be deposited on both sides of the cell, resulting in a reduction in the movement range, or even the stasis of the slider. The deposition of aged hydrates may cause the slider to be stuck directly. The total length of the cell was 200 mm. In the rocking period, the total movement range for the slider was ~193 mm. Based on this, the anti-agglomerating performance of AA/KHI were divided into three levels: A, B and C, from good to poor, as shown in Figure 3. If the slider is blocked, it indicates that the anti-agglomerating performance of AA/KHI is grade C. During the whole experiment, if the precipitation of hydrate aggregates led to a reduction in the slider movement range but no blockage, the anti-agglomerating performance of AA/KHI could be classified as grade B. If the slider was always moving freely through the cell throughout the experiment, the anti-agglomerating performance of AA/KHI could be classified as grade A.

### 3.2. Kinetic Inhibition

The kinetic inhibition performance was evaluated by measuring the subcooling degree and induction time. The subcooling degree (Δ*T*) is the difference between the hydrate nucleation temperature and the equilibrium temperature under the same pressure.

(9) $$\Delta T=T_{e}-T_{0}$$

where Δ*T* is the subcooling degree; °C; *T_e_* is the equilibrium temperature, which is calculated from system pressure in real time; °C; *T*_0_ is the hydrate nucleation temperature, °C.

Hydrate induction time was defined to be the difference between the nucleation time and the time when the system condition reached the equilibrium state. Therefore, the induction time (*t*) is the difference between *t*_1_ and *t*_0_, i.e.,

(10) $$t=t_{1}-t_{0}$$

where *t*_0_ is the time when the system temperature reaches hydrate equilibrium temperature, h; *t*_1_ is the time when the hydrate starts to form, h.

In order to exclude the effect of the randomness of hydrate nucleation on the classification criteria, the nucleation conditions in the water–oil system with 80% water cut were tested several times. The subcooling degree of the water–oil system is between 1.2–2 °C, and the induction time is between 0.5–1 h. With this as the boundary, the effects of AA/KHI on hydrate nucleation are divided into non-inhibition and inhibition, as shown in Table 2.

## 4. Anti-Agglomerating and Kinetic Inhibiting Performance of AA-1 and AA-2

The effects of gas–liquid ratio and water content, respectively, on the anti-agglomerating performance of AA were experimentally studied.

### 4.1. Effect of Water Cut

**Figure 4:**
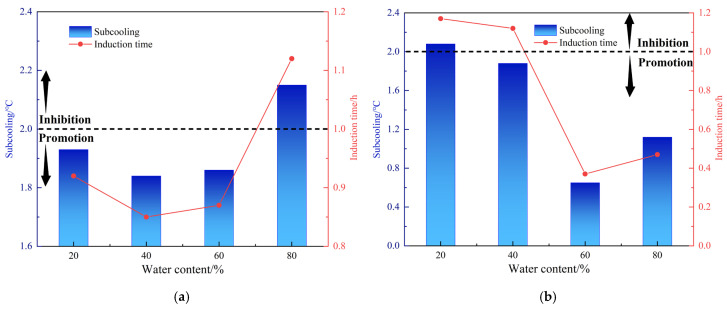
Effect of water content on the kinetic inhibiting performance of AA-1 and AA-2 (AA dosage: 2.5 wt%). (**a**) AA-1; (**b**) AA-2.

The change in water cut will affect the continuity of the oil–water two-phase distribution in the system, thereby affecting the stability of the oil–water emulsion. In this study, the inhibiting performance of the amide AA on the formation and agglomeration of methane hydrates was studied. In the experiment, the water content was the percentage of the water volume to the total volume of the liquid phase. The initial pressure was 8 MPa and the initial temperature was 16 °C. The rocking cell was cooled to 2 °C at a constant rate to form hydrate. The total volume of the liquid phase was kept at 100 mL, the water content of the system was changed by changing the volume of water and oil, and the AA/KHI content was calculated according to the water mass. Table 3 and Figure 4 show the inhibiting effect of two AAs on hydrates in oil, gas and water systems at 20–80% water cut. The results show that AA-1 is less affected by the water cut, the hydrate agglomeration avoidance can be achieved at the water cut of 20–80% and the anti-agglomerating performance is grade A. The water cut has a great influence on the anti-agglomerating performance of AA-2, and its anti-agglomerating performance increases with the increase in water cut. The anti-agglomerating performance reaches grade A at 80% water cut. According to the results, AA-1 and AA-2 are suitable for hydrate control at high water content. In terms of kinetic inhibiting performance, the AA-1 can only inhibit hydrate nucleation at 80% water cut. The maximum subcooling degree of the oil–water system reaches 2.15 °C, and the maximum induction time is 1.12 h. The AA-1 generally exhibit a non-inhibition effect on hydrate nucleation at a lower water cut (≤60%), and the maximum hydrate volume percentage is 23.27%. For AA-2, the water cut had a significant effect on the nucleation inhibition effect. AA-2 could only inhibit the hydrate nucleation at 20% water cut, with the subcooling degree of 2.08 °C and the induction time of 1.17 h. With the increase in water cut, AA-2 can promote hydrate nucleation. The minimum subcooling degree is 0.65 °C and the minimum induction time is 0.37 h. The hydrate volume percentage ranges from 11.32 to 20.73%.

### 4.2. Effect of Gas–Liquid Ratio

**Figure 5:**
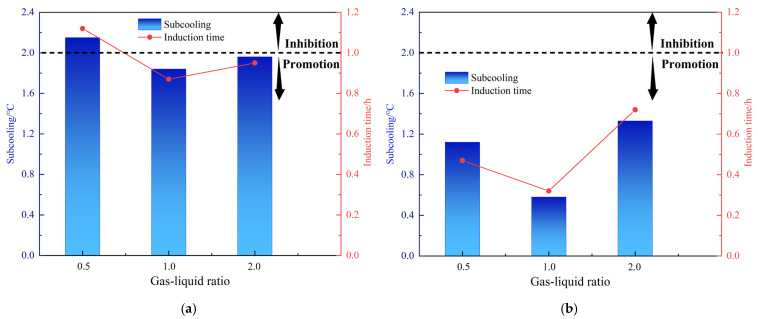
Effect of gas–liquid ratios on the kinetic inhibiting performance of AA-1 and AA-2 (AA dosage: 2.5 wt%). (**a**) AA-1; (**b**) AA-2.

The volume of the rocking cell is 150 mL. For evaluations, the volume ratio of gas phase to liquid phase is changed by changing the amount of liquid. In addition to the previous experiments in which the liquid phase is 100 mL and the gas–liquid ratio is 1:2, at the same water cut of 80% and initial pressure of 8 MPa, the volume of liquid phase was changed to 75 mL and 50 mL, corresponding to gas–liquid ratios of 1:1 and 2:1. Figure 5 and Table 4 show the performance of the two AAs at different gas–liquid ratios. The anti-agglomerating performance of AA-1 and AA-2 showed a decreasing trend when the gas–liquid ratio was increased. As the gas–liquid ratio increased, the mass transfer effect of the system is enhanced. The nucleation of hydrates was promoted and the hydrate volume percentage increased significantly. As the gas–liquid ratio increased to 2:1, both AAs lost their performance, and the effect dropped from grade A to grade C. At a gas–liquid ratio of 1:2, the subcooling degree of AA-1 is 2.15 °C and the induction time is 1.12 h and at a gas–liquid ratio of 2:1, the subcooling degree is 1.96 °C and the induction time is 0.95 h. AA-2 had no inhibition ability at the different gas–liquid ratios (1:2, 1:1 and 2:1), but had a strong ability to promote hydrate nucleation when the gas–liquid ratio was 1:1. The subcooling degree was only 0.58 °C, and the induction time was only 0.32 h. AA-2 generally shows a non-inhibition effect on hydrate formation, while AA-1 does not show an apparent kinetic effect.

## 5. Anti-Agglomerating and Kinetic Inhibiting Performance of AA-1/AA-2+PVP

**Figure 6:**
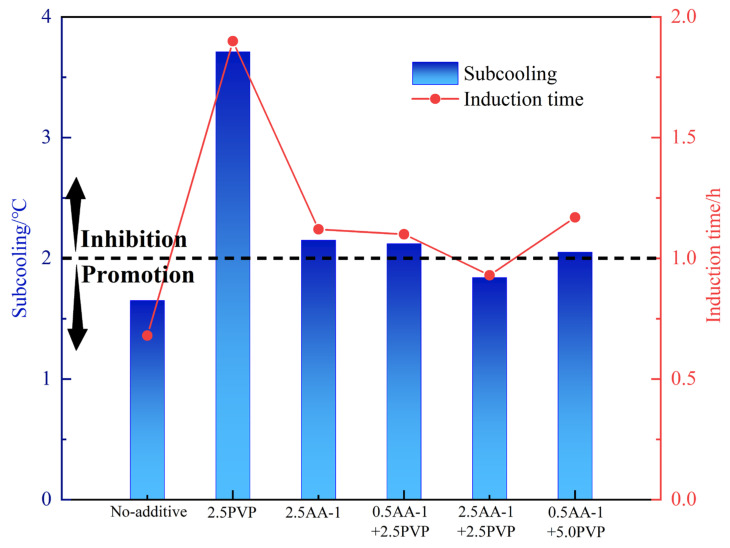
Inhibition performance of 2.5 wt% kinetic inhibition of AA-1+ at different concentrations.

**Figure 7:**
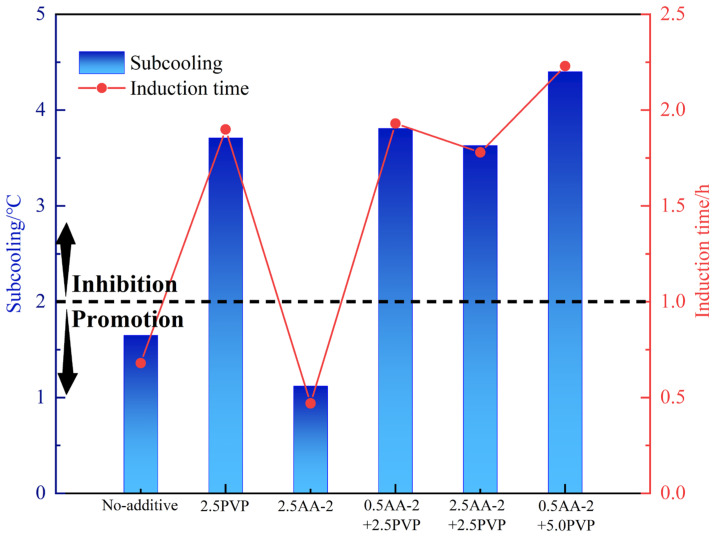
Kinetic inhibition performance of 2.5 wt% kinetic inhibition of AA-2+ at different concentrations.

The mutual effect of KHIs and AA may significantly affect the overall performance of the inhibitors. Accordingly, at 80% water content, the anti-agglomerating and kinetic inhibiting performance of AAs+PVP of different concentrations were evaluated. The results of the experiment are shown in Table 5 and in Figure 6 and Figure 7. PVP exhibited good compatibility with AA-2. With PVP, the anti-agglomerating performance of AA-2 can be kept as grade A, which means that hydrate is evenly dispersed in the water–oil emulsion. Notably, 2.5 wt% PVP could effectively retard hydrate nucleation in an oil–water system with 80% water cut. At the same anti-agglomerating performance, the addition of PVP enhanced the kinetic inhibition performance of AA-2. And with the increase in PVP concentration to 5.0 wt%, the inhibiting performance was elevated. At 5.0 wt% PVP concentration, the inhibition effect of the mixed LDHIs was found to be the strongest. The subcooling degree was increased by 3.28 °C compared with the single AA-2 system. The induction time was prolonged by 1.76 h. However, the kinetic inhibiting effect of the mixed PVP and AA-1 is weaker than that of AA-1-only system. At the PVP concentration of 2.5 wt%, the anti-agglomerating performance of the AA-1 was reduced to grade B. Overall, the use of AA-2+PVP can not only ensure the dispersion of hydrates to form a flowing slurry, but also prolong the hydrate nucleation time and slow down their formation. Therefore, AA-2+PVP may be a better option to reduce the risk of hydrate blockage.

For the inhibition mechanism, the different performances of mixtures is strongly dependent on the synergistic or competitive adsorption of PVP and AAs on hydrate surfaces. PVP inhibits hydrate nucleation and growth by the adsorption barrier and the disturbance of the water–molecule framework. After hydrate formation, AAs are adsorbed onto the hydrate surface to retard the agglomerating process. AA-1 and PVP may compete to adsorb on a hydrate surface. The initial adsorbed PVP may disturb the AA adsorption. Therefore, the anti-agglomerating performance was reduced. AA-2 has two amide groups and ammonium heads, and the adsorption may be stronger. Thus, both PVP and AA molecules can adsorb on hydrates to form a barrier. Keeping grade A anti-agglomerating performance, the mixture can prolong the induction time. The PVP and AA-2 exhibit a synergistic effect.

## 6. Conclusions

In this study, the effects of two amide hydrate anti-agglomerants on methane hydrate formation and agglomeration were determined by using a high-pressure hydrate rocking cell. Subsequently, the anti-agglomerants and PVP were mixed, and the anti-agglomerating and kinetic inhibition performance under different water contents and gas–liquid ratio conditions were determined. An evaluation method for LDHIs performance was established.

(1) At different water contents, it was found that AA-1 inhibits hydrate nucleation only at 80% water cut, while AA-2 inhibits hydrate nucleation at 20% water cut. The anti-agglomerating performance of AA-1 can reach grade A under different water cuts. However, the agglomeration inhibition performance of AA-2 is greatly affected by the water cut. The anti-agglomerating performance is enhanced with the increase in water cut, and grade A performance can be achieved only at 80% water cut.
(2) As the elevation of gas volume, gas–liquid exposure in the reactor increases, which promotes hydrate formation. The volume percentage of hydrates formed in water–oil also increases. With the increase in gas–liquid ratio, the agglomeration inhibition performance was gradually weakened. At a gas–liquid ratio of 2:1, AA-1 and AA-2 completely failed.
(3) AA-2 showed good adaptability with PVP, and the 5.0 wt% PVP + 2.5 wt% AA-2 mixture had the strongest inhibition effect. The subcooling degree reached 4.4 °C, the induction time was 2.23 h, and the anti-agglomeration performance reached grade A.

## Figures and Tables

**Table 1:** Specifications and sources of the chemicals used in the experiments.

Chemical	Formula	Specification	Factory
High-purity methane	CH_4_ (74-82-8)	99.999%	Qingdao Xinkeyuan Gas Co., Ltd. (Qingdao, China)
No. 5 white mineral oil	C_15_H_24_ (8042-47-5)	Industrial grade	Mojiezuo Petrochemical (Shang-hai) Co., Ltd. (Shanghai, China)
Purified water	H_2_O (7732-18-5)	18 MΩ·cm	Lab-made (Henan, China)
Petroleum ether	C_6_H_14_ (64742-82-1)	Spectrally pure	Shanghai Aladdin Biochemical Technology Co., Ltd. (Shanghai, China)
PVP	(C_6_H_9_NO)_n_ (9003-39-8)	MW = 10,000	Shanghai Macleane Biochemical Technology Co., Ltd. (Shanghai, China)
Coconut amidopropyl dimethylamine (AA-1)	C_17_H_20_N_4_O_2_ (68140-01-2)	Industrial grade	Shanghai Yincong New Material Technology Co., Ltd. (Shanghai, China)
Propylene bis (octadecylamidopropyl dimethylammonium chloride) (AA-2)	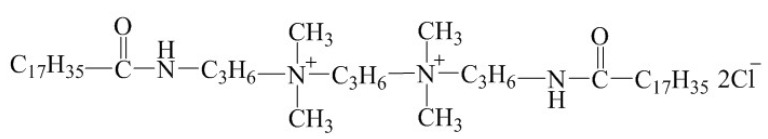	Industrial grade	Zhengzhou Yihe Fine Chemicals Co., Ltd. (Henan, China)

**Table 2:** Criteria for discriminating the inhibiting performance of KHIs.

Kinetic Effects	Hydrate Formation Conditions in Oil–Water Systems	
Inhibition	Subcooling ≥ 2 °C	Induction ≥ 1 h
Non-inhibition	Subcooling < 2 °C	Induction < 1 h

**Table 3:** Summary of the effect of the water cut on the anti-agglomerating and kinetic inhibiting performance of AA-1 and AA-2.

Sample	Water Cut /%	Subcooling /°C	Induction /h	Nucleation Inhibition	Hydrate Volume Fraction/%	Anti-Agglomerating
AA-1 (2.5 wt%)	20	1.93	0.92	Non-inhibition	23.27	A
	40	1.84	0.85	Non-inhibition	21.41	A
	60	1.86	0.87	Non-inhibition	20.36	A
	80	2.15	1.12	Inhibition	17.86	A
AA-2 (2.5 wt%)	20	2.08	1.17	Inhibition	11.64	C
	40	1.88	1.12	Non-inhibition	11.32	C
	60	0.65	0.37	Non-inhibition	20.73	B
	80	1.12	0.47	Non-inhibition	18.27	A

**Table 4:** Summary on the effect of gas–liquid ratios on the anti-agglomerating and kinetic inhibiting performance of AA-1 and AA-2.

Sample	Gas–Liquid	Subcooling	Induction	Nucleation	Hydrate Volume	Aniti-Agglomerating
	Ratio	/°C	/h	Inhibition	Fraction/%	
AA-1 (2.5 wt%)	0.5	2.15	1.12	Inhibition	17.86	A
	1	1.84	0.87	Non-inhibition	32.02	B
	2	1.96	0.95	Non-inhibition	51.83	C
AA-2 (2.5 wt%)	0.5	1.12	0.47	Non-inhibition	18.27	A
	1	0.58	0.32	Non-inhibition	33.08	B
	2	1.33	0.72	Non-inhibition	33.33	C

**Table 5:** Inhibition effect of amide anti-agglomerants + PVP composite hydrate inhibitor on hydrates in an oil–water system with 80% water cut.

Sample	Subcooling	Induction	Nucleation	Hydrate Volume	Aniti-Agglomerating
	/°C	/h	Inhibition	Fraction/%	
No-additive	1.65	0.68	——	10.63	C
2.5 wt%PVP	3.71	1.90	Inhibition	13.38	A
2.5 wt% AA-1	2.15	1.12	Inhibition	17.86	A
2.5 wt% AA-1+0.5 wt%PVP	2.12	1.10	Inhibition	18.49	A
2.5 wt% AA-1 + 2.5 wt%PVP	1.84	0.93	Non-inhibition	18.03	B
2.5 wt% AA-1 + 5.0 wt%PVP	2.05	1.17	Inhibition	18.87	A
2.5 wt% AA-2	1.12	0.47	Non-inhibition	18.27	A
2.5 wt% AA-2 + 0.5 wt%PVP	3.81	1.93	Inhibition	17.5	A
2.5 wt% AA-2 + 2.5 wt%PVP	3.63	1.78	Inhibition	18.15	A
2.5 wt% AA-2 + 5.0 wt%PVP	4.4	2.23	Inhibition	17.82	A

## Data Availability

Data are listed in the article.
